# Transcriptome analysis of HPV-induced warts and healthy skin in humans

**DOI:** 10.1186/s12920-020-0700-7

**Published:** 2020-03-09

**Authors:** Laith N. AL-Eitan, Amneh H. Tarkhan, Mansour A. Alghamdi, Firas A. Al-Qarqaz, Hadeel S. Al-Kofahi

**Affiliations:** 1 Department of Applied Biological Sciences, Jordan University of Science and Technology, Irbid, 22110 Jordan; 2 Department of Biotechnology and Genetic Engineering, Jordan University of Science and Technology, Irbid, 22110 Jordan; 3 Department of Anatomy, College of Medicine, King Khalid University, Abha, 61421 Saudi Arabia; 4 Genomics and Personalized Medicine Unit, College of Medicine, King Khalid University, Abha, 61421 Saudi Arabia; 5 Department of Internal Medicine, Jordan University of Science and Technology, Irbid, 22110 Jordan; 6 Division of Dermatology, Department of Internal Medicine, King Abdullah University Hospital, Jordan University of Science and Technology, Irbid, 22110 Jordan

**Keywords:** HPV, Cutaneous warts, RNA-sequencing, Transcriptome

## Abstract

**Background:**

The human papillomaviruses (HPV) are a group of viruses that, depending on the strain, can cause cancer or the formation of benign growths known as warts. Scarce information exists with regard to the genetic nature of non-genital cutaneous warts induced by the human papillomavirus (HPV).

**Methods:**

The main purpose of this study is to investigate the differences between the gene expression profiles of common warts and healthy skin in HPV-positive individuals by RNA sequencing on the Illumina HiSeq 2500. After obtaining shave biopsies of common warts and healthy skin from twelve Arab males, we were able to analyze the transcriptomes of 24 paired cases and controls.

**Results:**

Common warts were found to possess a highly significant and unique molecular signature. Many of the most up-regulated (*KRT16*, *EPGN*, and *ABCG4*) and down-regulated genes (*C15orf59*, *CYB561A3*, and *FCGRT*) in warts were the subject of little investigation in the published literature. Moreover, the top 500 differentially expressed genes were found to be associated with immune and autoimmune pathways, such as the neutrophil degranulation, toll-like receptor 7/8 (TLR 7/8) cascade, toll-like receptor 9 (TLR9) cascade, and toll-like receptor 10 (TLR10) pathways, among others.

**Conclusions:**

Our findings are particularly important because they serve as the most comprehensive to date with regard to the modulation of human skin gene expression by HPV infection.

## Background

The human papillomavirus (HPV) is a DNA virus that has been associated with many diseases and is the definitive cause of virtually all cervical cancer cases [1]. Nearly two hundred types of HPV have been characterized, and these types are classified as high-risk or low-risk depending on their potential to cause cancerous or benign lesions, respectively [2]. 4.5% of new cancer cases worldwide are caused by high-risk HPV infection, but low-risk types often manifest in the form of warts [3, 4]. Common warts (*Verruca vulgaris*) are by far the most prevalent type of wart, making up 70% of all non-genital cutaneous warts, and are thought to be benign in nature [5, 6].

Human skin is composed of two layers, the epidermis and the dermis, and HPV exclusively targets the lowermost epidermal layer, the stratum basale, in order to establish a persistent infection, after which it hijacks the keratinocyte differentiation process for the purpose of the productive viral life cycle [7, 8]. Correspondingly, HPV biology differs in basal and suprabasal keratinocytes, the latter of which are involved in different stages of epithelial differentiation [9]. Keratinocytes make up 90% of all epidermal cells, and perturbations to their differentiation make the skin more susceptible to infection and disease [10]. Currently, there is no cure for HPV itself, and its treatment focuses on the alleviation of clinical symptoms until the infection is naturally cleared by the immune system [11, 12]. However, there is no conclusive evidence as to whether the virus is completely eliminated from the body or simply lowered to undetectable levels [12].

While much research has been dedicated to the genetics of HPV-associated cancers, there is a dearth of information concerning the genetic background of non-genital cutaneous warts, which indicates a pressing need to better understand their etiology. Therefore, the aim of the present study is to provide a genome-wide comparison of the transcriptomes of common warts and normal skin using next-generation sequencing.

## Methods

### Subjects

The present study was approved by the Institutional Review Board (IRB) at Jordan University of Science and Technology (JUST) (Ref. 13/105/2017). All procedures performed in this study were in compliance with the IRB’s human research ethics protocol and were performed at King Abdullah University Hospital in Irbid, Jordan. Twelve Arab males presenting with common warts were recruited from the general population after giving written informed consent. Clinical diagnosis was established by a dermatologist based on typical clinical features for warts, especially keratotic surfaces and black thrombosed capillaries or pin-point bleeding upon paring. Epidermal tissue samples from warts (*n* = 12) and normal skin (controls) (n = 12) were obtained via a superficial shave biopsy after the application of a local anesthetic. Normal skin tissue samples were procured from a site adjacent to the area of the wart excision. Most samples came from the dorsal aspect of the hands (*n* = 20), while a minority were obtained from the forehead (n = 2) and the dorsal aspect of the foot (n = 2). Biopsies were immediately stored in microcentrifuge tubes containing RNA-SafeGuard reagent (GMbiolab Co., Ltd., Taiwan) and refrigerated at − 20 °C until further processing.

### RNA extraction

Total RNA was extracted from the epidermal samples by means of an RNeasy Mini Kit (Qiagen, Germany), and optional on-column DNase digestion was performed. The quality (260/280), quantity (ng/ml), and RNA integrity numbers (RIN) of the purified RNA was then ascertained using the Biotek PowerWave XS2 Spectrophotometer (BioTek Instruments, Inc., USA) and the Agilent Bioanalyzer (Agilent, USA). All samples selected for sequencing had a RIN value of greater than 7.5.

### RNA sequencing (RNA-Seq)

Samples were shipped on dry ice to the Melbourne node of the Australian Genome Research Facility (AGRF). RNA-Seq was carried out on the Illumina HiSeq 2500 according to the manufacturer’s protocol. Image analysis was performed in real time by the HiSeq Control Software (HCS) v2.2.68 and Real Time Analysis (RTA) v1.18.66.3 running on the instrument computer. Then, the Illumina bcl2fastq 2.20.0.422 pipeline was used to generate the sequence data. All generated data met AGRF quality standards.

### Bioinformatics analysis

The sequence reads from all 24 samples were analyzed and screened according to AGRF quality control measures. edgeR version 3.22.3 was used to detect and quantify differential expression of digital gene expression data (counts of reads mapped for each gene). Unsupervised clustering of the 24 samples was carried out using a multi-dimensional scale (MDS) plot for raw data and normalized data. The trimmed mean of M-values (TMM) normalisation method was performed to overcome library size differences among samples. The TMM normalisation was done on feature counts data, the latter of which was then transformed to log cpm (counts per million) values to normalize for the different sequencing depths for each sample (Supplementary Figure S1). A generalised linear model was then used to quantify the differential expression between the groups.

Functional enrichment analysis of DE genes was performed using the Database for Annotation, Visualization and Integrated Discovery (DAVID) version 6.8 (Leidos Biomedical Research, Inc., USA). Pathways and network patterns associated with DE genes were analyzed using Reactome version 65 (https://reactome.org) and corrected for false discovery rate (adjusted *p*-value) using the Benjamani-Hochberg method. The signaling network of DE genes was explored using the Signaling Network Open Resource 2.0 (Signor). The ReactomePA pathway analyser was used to enrich for the list of pathways associated with the top 500 DE genes. Next, ComplexHeatmap version 1.18.1 was used to generate heatmaps for each comparison showing the clustering of each sample present in the comparison using the top 50 most differentially expressed (DE) genes for that comparison.

Moreover, cell type enrichment analysis was performed for infiltrating immune cell genes with FDR > 0.05. ClustVis (http://biit.cs.ut.ee/clustvis/) was used to create a heatmap that compared between the infiltrating immune cell genes in warts and normal skin. In addition, we performed immune cell type enrichment analysis data for genes with FDR > 0.05. The analysis was conducted using the xCell webtool (http://xCell.ucsf.edu/) and xCell gene signatures as references of immune cells [14]. The generated adjusted cell type enrichment scores were used to create a heatmap comparing infiltrating immune cell genes in warts and normal skin using the ClustVis webtool (http://biit.cs.ut.ee/clustvis/).

Lastly, HPV subtypes were identified from the RNA-seq data by utilizing the web-based Viral Genome-Targeted Assembly Pipeline (VirusTAP) [15]. VirusTAP performs quality trimming and subtracts unnecessary reads such as rRNA, bacterial genomes, and the host genome. Afterwards, de novo transcriptome assembly and megablast homology search were performed of contigs against the NCBI database. Finally, the trimmed reads were mapped back to the assembled contigs.

## Results

### HPV genotyping

A total of 5 HPV subtypes were detected in the RNA-seq data, including HPV 57 (Alphapapillomavirus 4), HPV 2 (Alphapapillomavirus 4), HPV 27 (Alphapapillomavirus 4), HPV 4 (Gammapapillomavirus 1), and HPV XS2 (Alphapapillomavirus 2). Tables 1 and 2 show the results of HPV genotyping for the control and wart samples. Interestingly, no contigs related to virus sequences were found in one of the wart samples, 9 W, while one of the control samples, 4C, was shown to have HPV XS2.

**Table 1:** HPV typing and location of wart (W) samples

Patient	1 W	2 W	3 W	4 W	5 W	6 W	7 W	8 W	9 W	10 W	11 W	12 W
Site	Hand	Hand	Hand	Hand	Hand	Hand	Hand	Hand	Forehead	Hand	Hand	Foot
HPV type	2	57	XS2	27	57	4	27	2	-ve	2	57	57

*-ve* no contigs related to virus sequences were found*.*

**Table 2:** HPV typing and location of control (C) samples

Patient	1C	2C	3C	4C	5C	6C	7C	8C	9C	10C	11C	12C
Site	Hand	Hand	Hand	Hand	Hand	Hand	Hand	Hand	Forehead	Hand	Hand	Foot
HPV type	-ve	-ve	-ve	XS2	-ve	-ve	-ve	-ve	-ve	-ve	-ve	-ve

*-ve* no contigs related to virus sequences were found.

### Differentially expressed (DE) genes

**Fig. 1:**
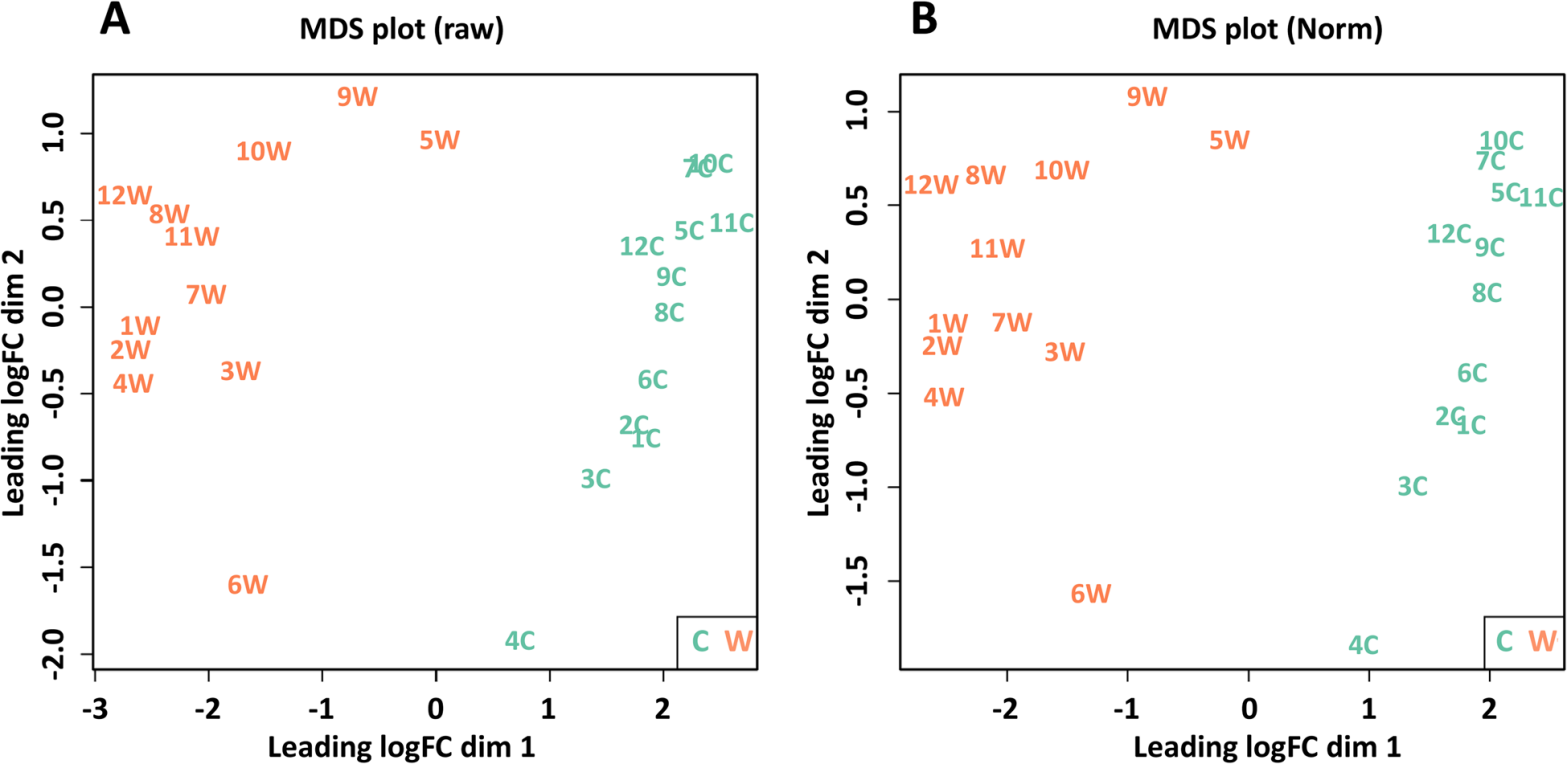
Multidimensional scaling (MDS) plot showing variation among samples based on (**a**) raw data and (**b**) normalized data. Each point represents 1 sample, and the distance between 2 points reflects the leading logFC of the corresponding RNA-Seq samples. The leading logFC (base 2 logarithm of fold change) is the average of the largest absolute logFC between each pair of samples. The plot dimension 1 (dim 1) illustrates that the control (C) and wart (W) samples form separate clusters with certain dispersion among samples

Unsupervised clustering revealed clear differences between the control (normal skin) and wart samples (Fig. 1). The wart samples were grouped together and separated from control samples based on the similarity of gene expression profiles. This confirms the paired nature of the samples. The wart samples appear more heterogeneous than the normal skin samples. However, sample 4C, which is the only control sample to contain an HPV type, was further away than the rest of the control samples. In addition, sample 6 W, which contained HPV 4, was located far from the rest of the clustered wart samples. This was to be expected as HPV4 is a Gammapapillomavirus species, while the rest of the identified HPV types were Alphapapillomaviruses. Although samples 9 and 12 came from the forehead and foot, respectively, they were located within each cluster alongside the samples obtained from the hand area.

**Fig. 2:**
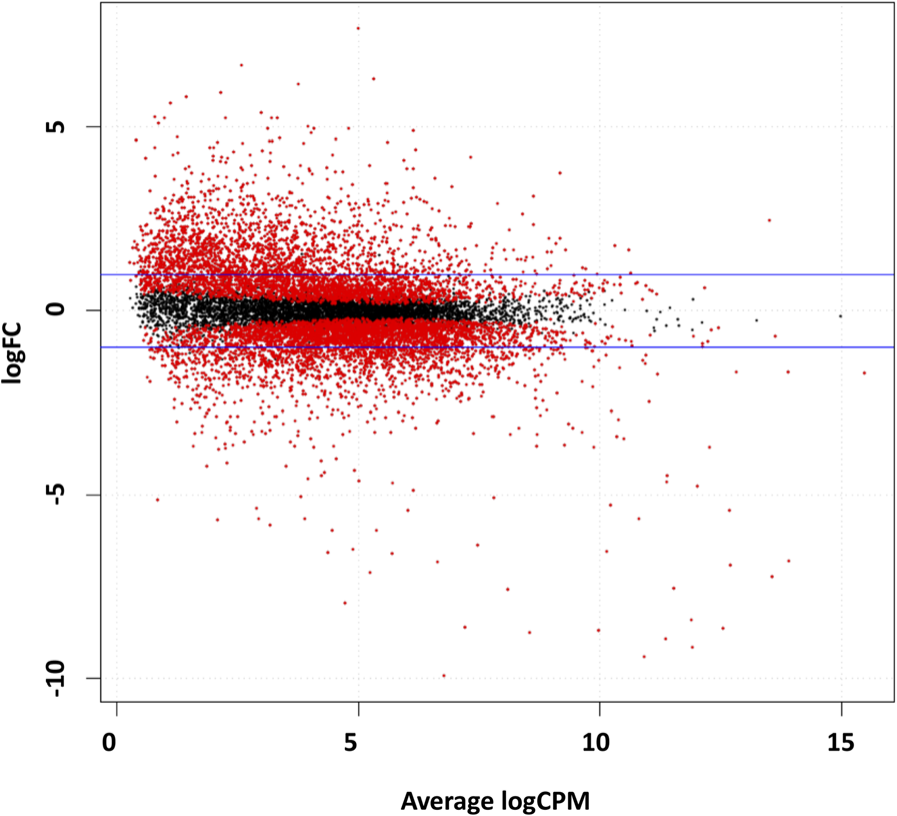
Smear plot of DE genes in warts. The logFC (base 2 logarithm of fold change) for each gene is plotted against the average abundance in logCPM (base 2 logarithm of counts per million). The two horizontal blue lines represent two fold changes. The red points are those with an AP of less than 0.05, while the black points are those with an AP of more than 0.05

**Fig. 3:**
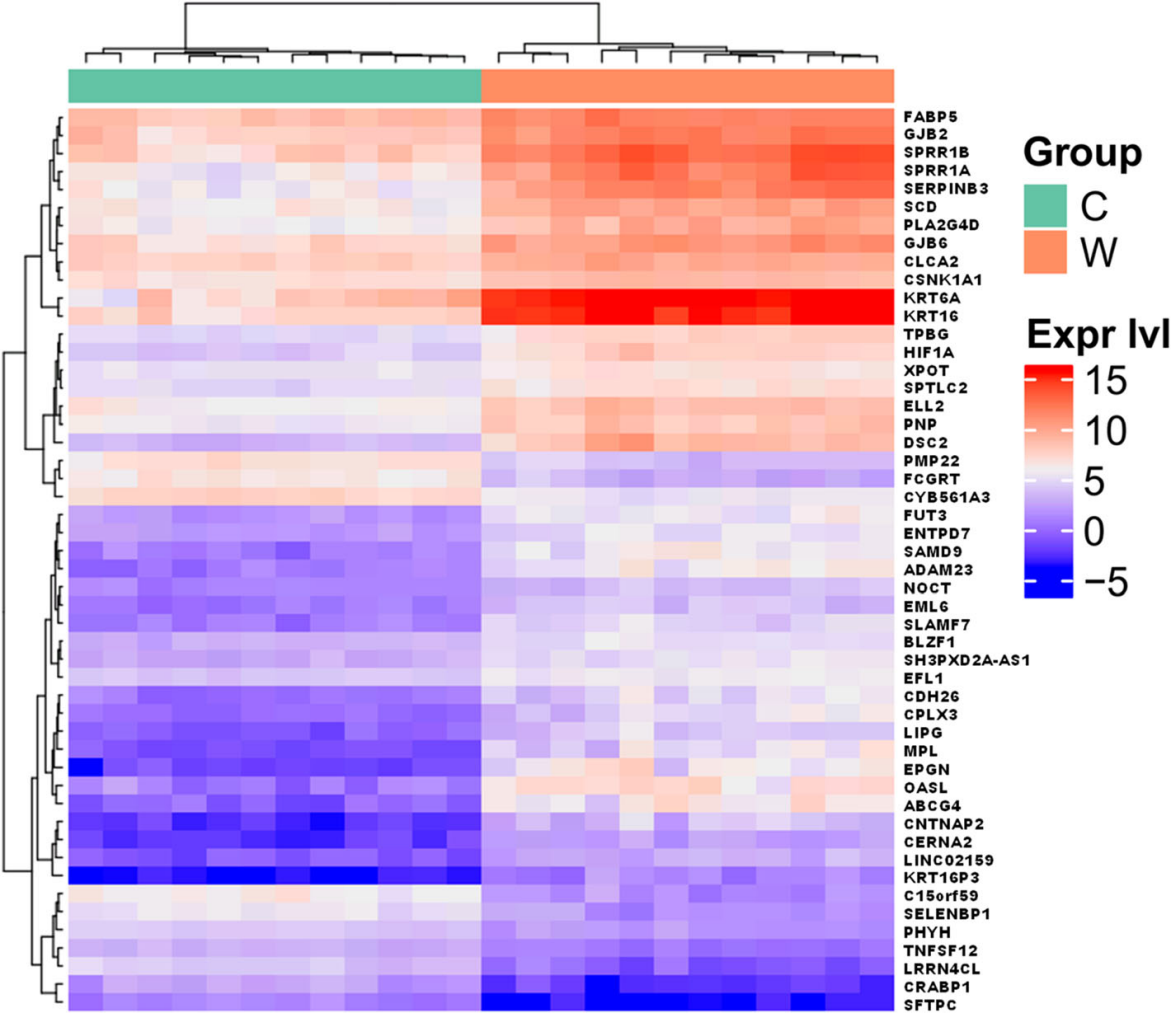
Heatmap of the top 50 DE genes between warts (W) and normal skin (C). The hierarchically clustered genes are represented by rows, and the samples are represented by columns, while the dendrograms and flat clusters are symbolized by the green and orange bars. Genes that have positively correlated logCPM values cluster together, as large positive correlations correspond to small distances. The red and blue colors indicate gene upregulation and downregulation, respectively

From among 13,574 tested genes, 8007 were shown to be DE (adjusted *p*-value (AP) < 0.05) between warts and normal tissue. The 8007 genes were then filtered out by fold change (FC), resulting in a total of 3140 DE genes (FC > 2) (Fig. 2). Of these DE genes, a total of 1732 (55%) and 1408 (45%) genes were found to be upregulated and downregulated, respectively (Fig. 2). Supplementary Table S1 lists the top 100 DE genes in warts, while Supplementary Figure S2 shows the overall heatmap of the top 500 most DE genes. In contrast to Supplementary Figure S2, Fig. 3 shows the hierarchal clustering heatmap for the top 50 most DE genes in warts and normal skin. The similarity in clustering patterns within each of the wart and control (normal skin) groups indicate that there are distinct expression profiles for warts.

### Pathway and functional enrichment analysis

**Fig. 4:**
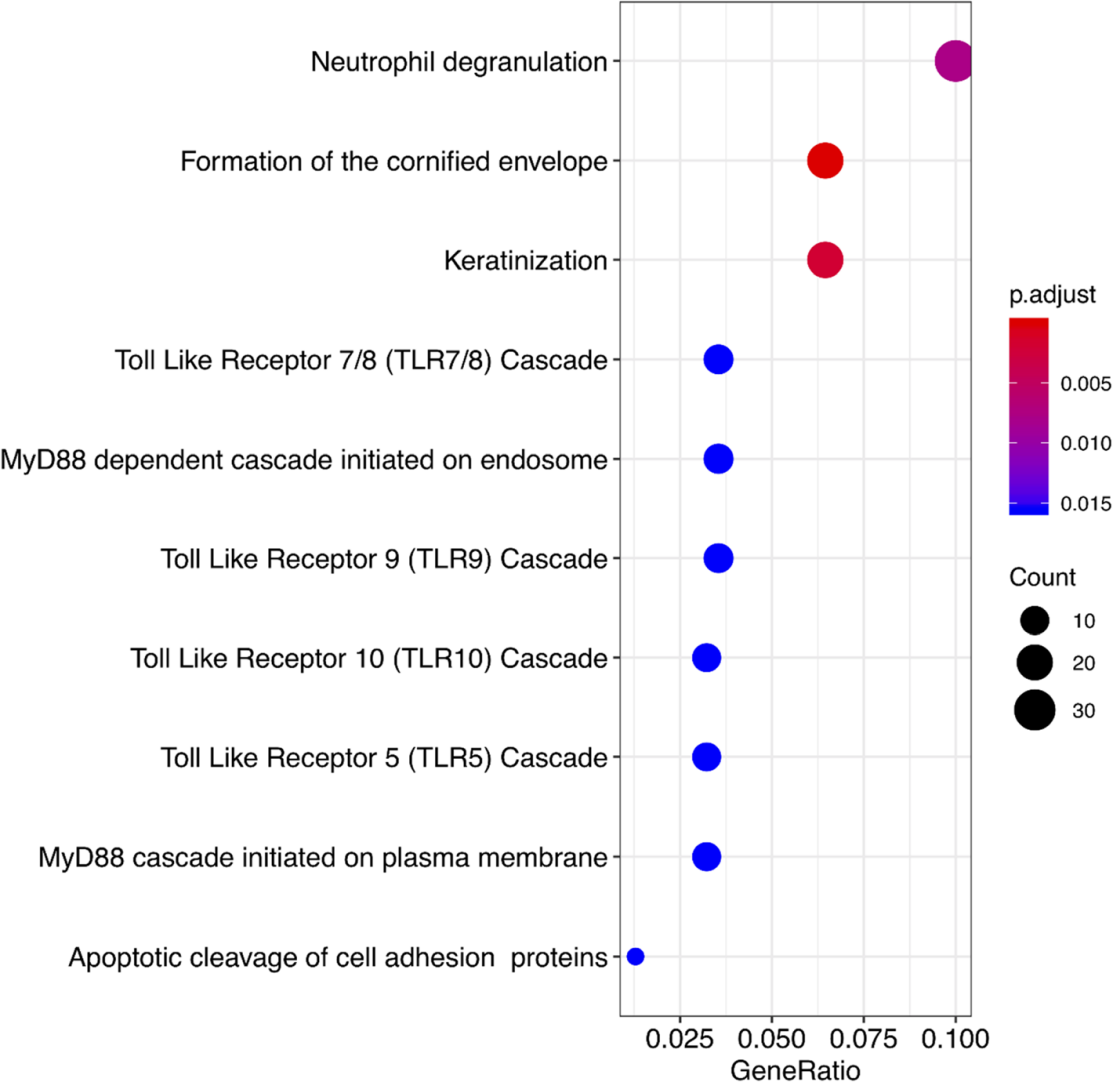
Top 10 pathways associated with the top 500 DE genes in terms of adjusted *p*-value

**Fig. 5:**
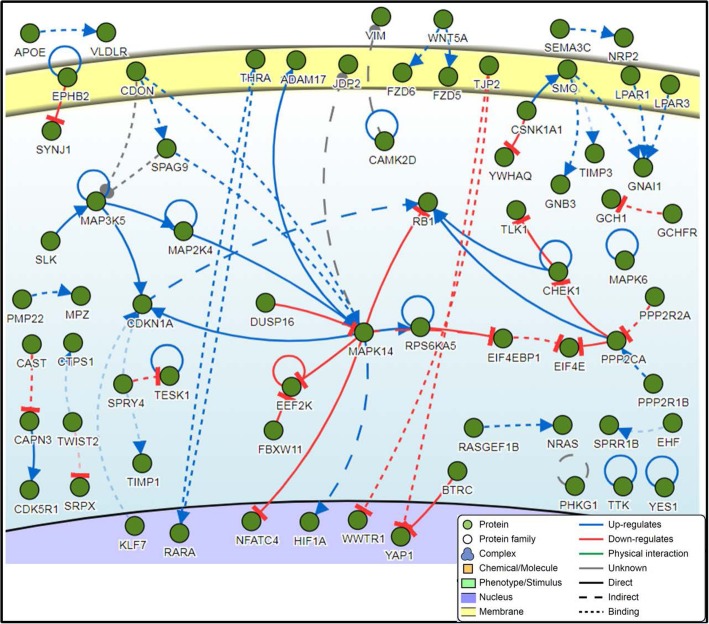
Pathway network generated from the top 1000 DE genes demonstrating the various interactions between proteins. Four genes have at least five connections (*MAPK14*, *PPP2CA*, *CDKN1A*, and *MAP3K5*)

Enrichment analysis of the DE genes revealed 71 functional annotation clusters, and the clusters with an enrichment score > 2 and GO term *p*-value < 0.05 are shown in Table S2. The top enriched terms include the cornified envelope, keratinization, keratinocyte differentiation, and peptide cross-linking. Moreover, by enriching for only the top 500 DE genes and using a cut-off p-value of 0.05, 10 pathways of interest were reported (Fig. 4). Pathway analysis of the top 1000 DE genes was performed in order to determine any significant gene-protein associations. It was found that the *CDKN1A* (logFC = − 1.18; AP = 0.00015), *MAPK14* (logFC = − 1.27; AP = 1.16 × 10^− 10^), *MAP3K5* (logFC = − 1.43; AP = 4.97 × 10^− 8^), and *PPP2CA* (logFC = − 1.2; AP = 8.21 × 10^− 8^) genes had the highest number of interactions with other DE genes in wart (Fig. 5). In fact, functional enrichment analysis showed that *CDKN1A* is involved in the protein serine/threonine kinase activity pathway, *MAPK14* is involved in the protein serine/threonine kinase and protein kinase activity pathways, and *MAP3K5* is involved in the protein phosphorylation, protein serine/threonine kinase, and protein kinase activity pathways.

**Fig. 6:**
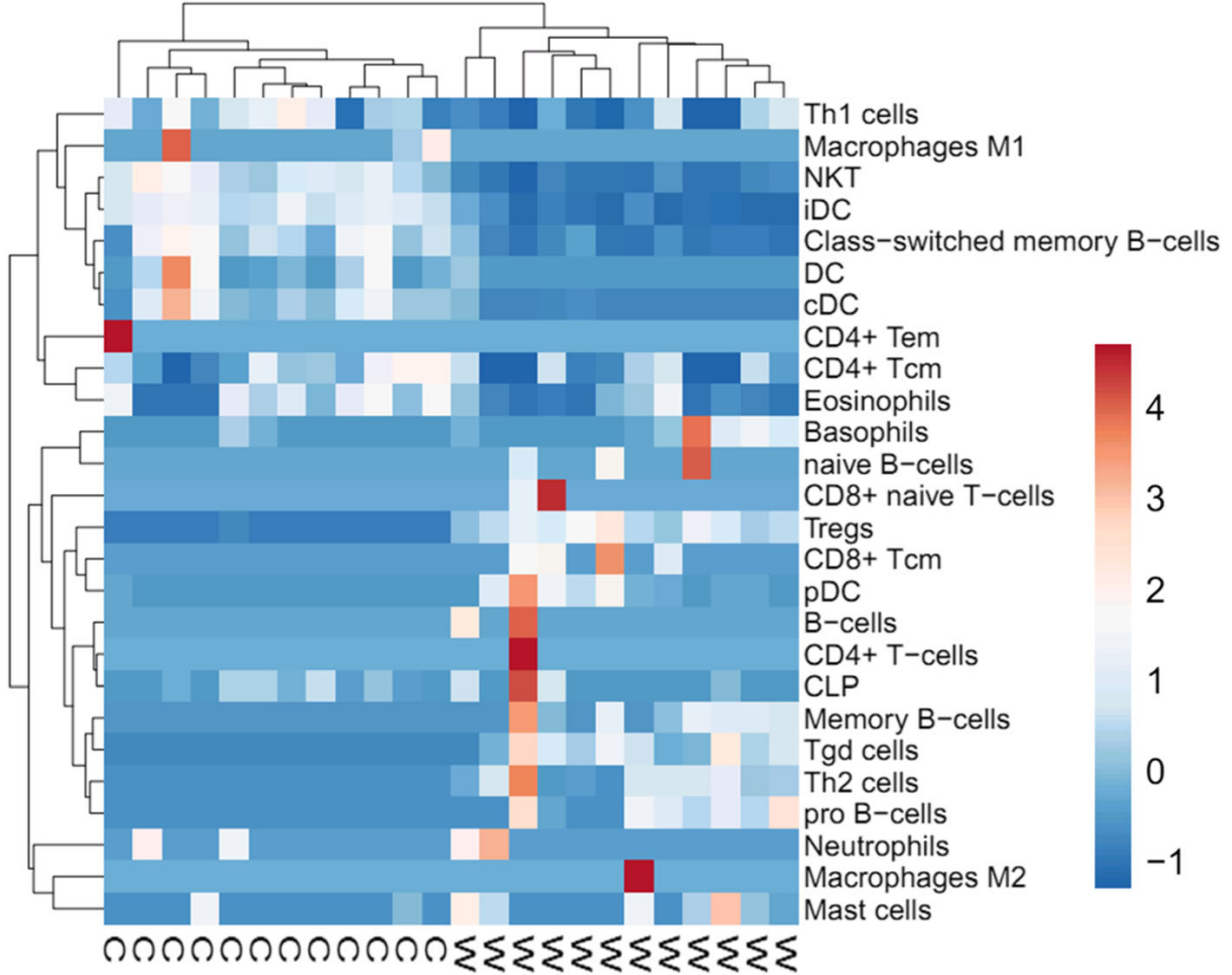
Heatmap of immune cell infiltrates expressed in warts (W) compared to normal skin samples (C). Both rows and columns are clustered using correlation distance and average linkage. The dendrogram at the top shows the samples; the dendrogram at the right shows different immune cell signatures. Blue color indicates lower expression of a particular immune cell signature and red color indicates higher expression

To identify the immune gene signature, we performed cell type enrichment analysis and heatmap clustering of the genes in the present study with an FDR cut-off value of 0.05. We found 26 immune cell populations which are differentially activated in warts compared to normal skin (Fig. 6). The immune cell signature dendrogram appears to be split between natural killer T (NKT) cells, immature dendritic cells (iDCs), class-switch memory B-cells, conventional dendritic *cells* (cDCs), *regulatory T cells* (Tregs), and gamma delta T (*Tgd*) *cells* with varying expression of these in the samples.

## Discussion

Although warts are themselves benign, HPV infection has been identified as the definitive cause of cervical cancer and has been associated with a number of other cancers and diseases [16]. As a result, the majority of peer-reviewed studies conducted on HPV infection have been in the context of cancer research. However, HPV has been detected in a wide plethora of skin conditions other than cancer, including actinic keratoses, epidermal cysts, lichen sclerosus, psoriatic plaques, seborrheic keratoses, and skin tags, and it has also been detected on healthy skin and plucked hairs [17]. This ubiquity of HPV in human physiology and pathology mandates that its role in non-genital cutaneous warts be elucidated.

With regard to upregulated genes, *KRT16* was found to be the most upregulated gene in warts. *KRT16* encodes for keratin 16, a type I cytokeratin that plays an essential role in the barrier function, innate immunity, and network signaling of the skin epidermis as well as the integrated stress response shared by all eukaryotes [18, 19]. Aberrant *KRT16* expression has been implicated in the as a number of inflammatory skin diseases as well as breast and pancreatic cancer [13, 20, 21]. In fact, *KRT16* expression was increased by two-fold in keratinocytes infected with high-risk HPV [22]. In addition, *EPGN* was shown to be the second most upregulated gene in the present study and encodes for epithelial mitogen, an epidermal growth factor whose biologic function was still unknown [23]. The role of the *EPGN* gene in keratinocytes and warts have not yet been considered.

In terms of downregulated genes, this study found that the chromosome 15 open reading frame 59 (*C15orf59*) is the most downregulated gene in warts. Very little is known about the *C15orf59* gene either in terms of its function or its role in disease. The second most down-regulated gene is the cytochrome b561 family member A3 (*CYB561A3*) gene, which is normally expressed intercellularly as well as at the cell membrane and was found to be down-regulated in sepsis [24]. Compared to the *C15orf59* and *CYB561A3* genes, much more is known about the third most down-regulated gene, Fc fragment of IgG receptor and transporter (*FCGRT*), which encodes for the neonatal Fc receptor and is normally expressed in human epidermal keratinocytes [25]. In cell lines infected with high-risk HPV, *FCGRT* expression on epithelial keratinocytes was found to modulate the uptake of IgG antibodies [26].

In this study, the top wart-associated pathways were involved in the immune system, pointing towards the effects of HPV infection on immune function. The most significantly associated pathway was neutrophil degranulation, a component of the innate immune system which was found to be a typical feature of inflammatory disorders such as asthma and acute lung injury [27]. The second and third most significantly associated pathways, formation of the cornified envelope and keratinization, are involved in the formation of wart hyperkeratosis, which in itself is a form of programmed cell death or apoptosis [28]. The toll-like receptor 7/8 (TLR7/8) cascade, the fourth most significantly associated pathway, plays an integral role in innate immunity, but its aberrant expression was significantly associated with persistent HPV16 infection [29]. In addition, the TLR9, TLR10, and TLR5 cascades were associated with the DE genes in our study, and they were similarly dysregulated in HPV infection and cervical cancer progression [29, 30]. Two of the top ten pathways involved the myeloid differentiation factor 88 (MyD88), a protein involved in innate immune function that was upregulated in HPV-transformed cervical cancer cell lines [31].

Moreover, the immune gene expression signature showed a list of immune cells which are differentially activated in warts compared to normal skin. Natural killer T (NKT) cells, a key component of the innate immune system, are important in killing certain tumour cells as well as virally infected cells, and they have been associated with HPV infection [32]. Similarly, regulatory T (Treg) cells are considered to aid in HPV growth by playing a role in immunosuppression [33]. Additionally, three dendritic cell (DC) subsets, namely immature dendritic cells (iDCs), conventional dendritic cells (cDCs) and plasmacytoid dendritic cells (pDCs), showed low activity in wart samples compared to normal skin. DCs have an essential role in initiating and regulating innate and adaptive immune responses to viral infection [34].

A pathway analysis of all 3140 DE genes found that four genes were central to the pathway: cyclin dependent kinase inhibitor 1A (*CDKN1A*), mitogen-activated protein kinase 14 (*MAPK14*), mitogen-activated protein kinase kinase kinase 5 (*MAP3K5*), and protein phosphatase 2 catalytic subunit alpha (*PPP2CA*). Our study showed that *CDKN1A* had the highest number of interactions with the other DE genes in this study and was itself downregulated in warts. The *CDKN1A* gene, which encodes for the cyclin-dependent kinase inhibitor p21, is a transcriptional target of the tumor suppressor protein p53 and functions to induce cell cycle arrest in the event of DNA damage [35, 36]. In the context of HPV type, it appears to be that the alphapapillomaviruses, including the low-risk HPV2 and HPV57 and the high-risk HPV15 and HPV18, target the p53-p21 pathway regardless of oncogenic potential [37, 38].

Additionally, *MAPK14* has been documented as having an important role in cell survival and apoptosis as well as differentiation and proliferation [39]. In fact, *MAPK14* plays a dual role in the skin, where it is responsible both for evoking an inflammatory reaction and for containing the aforementioned inflammation [40]. Moreover, *MAP3K5,* which is also known as apoptosis signal-regulating kinase 1 (*ASK1*), responds to inflammatory and physical stressors by activating the JNK and p38 pathways, and it plays a key role in hair regrowth in wounded skin [41]. Lastly, *PPP2CA* has been determined to play a critical role in the development of both the epidermis and the hair follicles [42].

Several strengths can be attributed to the present study that consolidate the significance and accuracy of its findings. All of our patients were Jordanian-Arab males ranging from 18 to 27 years old, and both the case (wart) and control (normal skin) biopsies were obtained from the same patient. None of the warts biopsied for this study have been modified by any type of treatment or medicine. Moreover, case and control biopsies for ten out of twelve patients in this study were carried out on the same area of the body, i.e. the hands, further reducing the amount of variability between subjects. Finally, all the warts included in this study were of the same type, i.e. *Verruca vulgaris.*

However, some limitations exist regarding the current study. Although only common warts (*Verruca vulgaris*) were investigated, HPV-induced warts are heterogeneous in nature, and several different HPV types have been associated with common warts, including HPV 2, 4, 7, 27, and 57 [43]. In addition, the HPV genome is itself highly heterogenous due to the fact that each HPV type has evolved to maximize viral fitness to its biological ecosystem within the host, i.e. niche adaptation [44, 45]. In the present study, HPV typing revealed that the investigated warts were caused by HPV types belonging to the Alphapapillomavirus 2 (HPV XS2), Alphapapillomavirus 4 (HPV 2/27/57), and Gammapapillomavirus 1 (HPV4) species. Another limitation is the fact that histological analysis of the wart samples was not possible, as the sample biopsies were small and entirely used up during the RNA extraction process. Since HPV biology differs between epidermal layers, this means that the present RNA-seq data contains a mixture of basal and suprabasal expression patterns. A final limitation is the relatively small sample size, but this is in line with the sample sizes of other RNA-seq studies, which included as little as four patients compared to our twelve [46].

## Conclusion

The present study served to clarify the genetic background of HPV infection in the context of non-genital cutaneous warts. The findings of the current study highlighted dysregulation of multiple genes which might play a critical role in wart formation, including *KRT16*, *EPGN*, *C15orf59*, *CYB561A3,* and *FCGRT*. Furthermore, several immune system related pathways were found to be associated with identified DE genes including neutrophil degranulation, myeloid differentiation factor 88, and toll-like receptors.

Our data also suggests that several genes involved in the immune response may play a critical role in facilitating the HPV infection process. Indeed, immune cell signature analysis showed different activity levels of multiple cells involved in the immune system including NKT, DC, and Treg cells. Many of the genes found to be implicated in common warts were not the subject of previous peer-reviewed investigation, suggesting future directions and lines of research targeting those individual genes as well as the expression patterns of individual HPV types.

## Supplementary information

**Additional file 1 Figure S1.** Distribution of gene expression values. Box plots show the distribution of log counts (A) before and (B) after normalization. **Figure S2.** Overall heatmap of the top 500 most DE genes. The hierarchically clustered genes are represented by rows, and the samples are represented by columns, while the dendrograms and flat clusters are symbolized by the green and orange bars. Genes that have positively correlated logCPM values cluster together, as large positive correlations correspond to small distances. The red and blue colors indicate gene upregulation and downregulation, respectively.

**Additional file 2 Table S1.** Top 100 DE genes in warts (FC > 2 and AP < 0.05) as sorted by the quasi-likelihood F test (QLF).

**Additional file 3 Table S2.** Functional annotation clustering of the DE genes only showing DE genes with a *p*-value < 0.05. BP stands for biological processes, MF for molecular function, CC for cellular component, and GO for gene ontology.

## Data Availability

The data generated over the course of the present study has been deposited to NCBI’s Gene Expression Omnibus (GEO) under the GEO accession number of GSE136347.

## Abbreviations

AGRF: Australian Genome Research Facility
BCC: Basal cell carcinoma
DE: Differentially expressed
DNA: Deoxyribonucleic acid
FC: Fold change
HPV: Human papilloma virus
IL: Interleukin
IRB: Institutional Review Board
PCR: Polymerase chain reaction
RIN: RNA Integrity Number
RNA: Ribonucleic acid
RNA-Seq: RNA-Sequencing
SNP: Single nucleotide polymorphism
