# Supplementary material for: Transcriptome analysis of HPV-induced warts and healthy skin in humans
Source: BMC Med Genomics. 2020 Mar 9;13:35. doi: 10.1186/s12920-020-0700-7 (PMC7063766; doi:10.1186/s12920-020-0700-7)
Supplement: Supplementary file 1 — Additional file 1 Figure S1. Distribution of gene expression values. Box plots show the distribution of log counts (A) before and (B) after normalization. Figure S2. Overall heatmap of the top 500 most DE genes. The hierarchically clustered genes are represented by rows, and the samples are represented by columns, while the dendrograms and flat clusters are symbolized by the green and orange bars. Genes that have positively correlated logCPM values cluster together, as large positive correlations correspond to small distances. The red and blue colors indicate gene upregulation and downregulation, respectively. [file 12920_2020_700_MOESM1_ESM.pptx]

## Slide 1
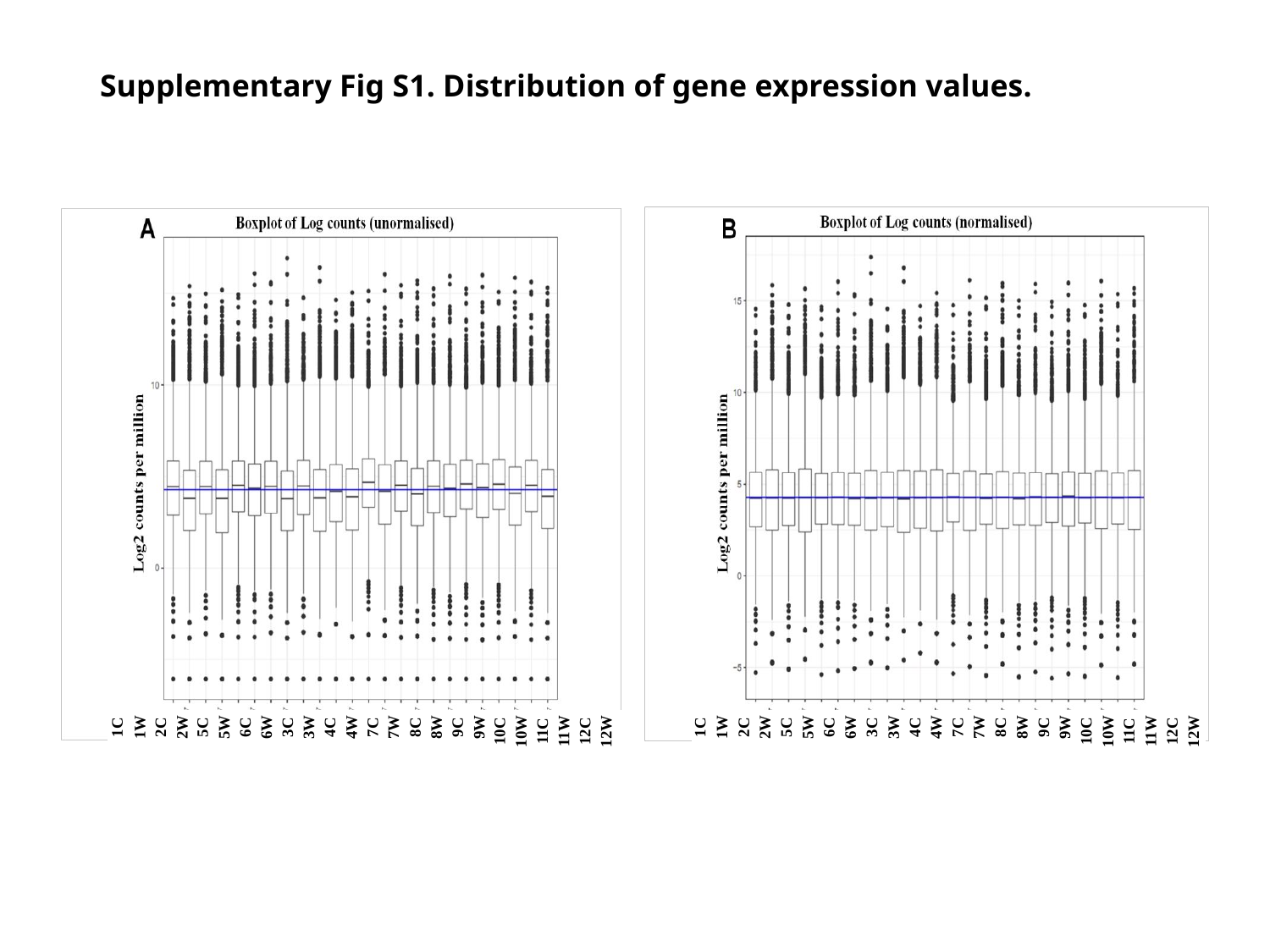

# Supplementary Fig S1. Distribution of gene expression values.
1C
1W
2C
2W
5C
5W
6C
6W
3C
3W
4C
4W
7C
7W
8C
8W
9C
9W
10C
10W
11C
11W
12C
12W
1C
1W
2C
2W
5C
5W
6C
6W
3C
3W
4C
4W
7C
7W
8C
8W
9C
9W
10C
10W
11C
11W
12C
12W

## Slide 2
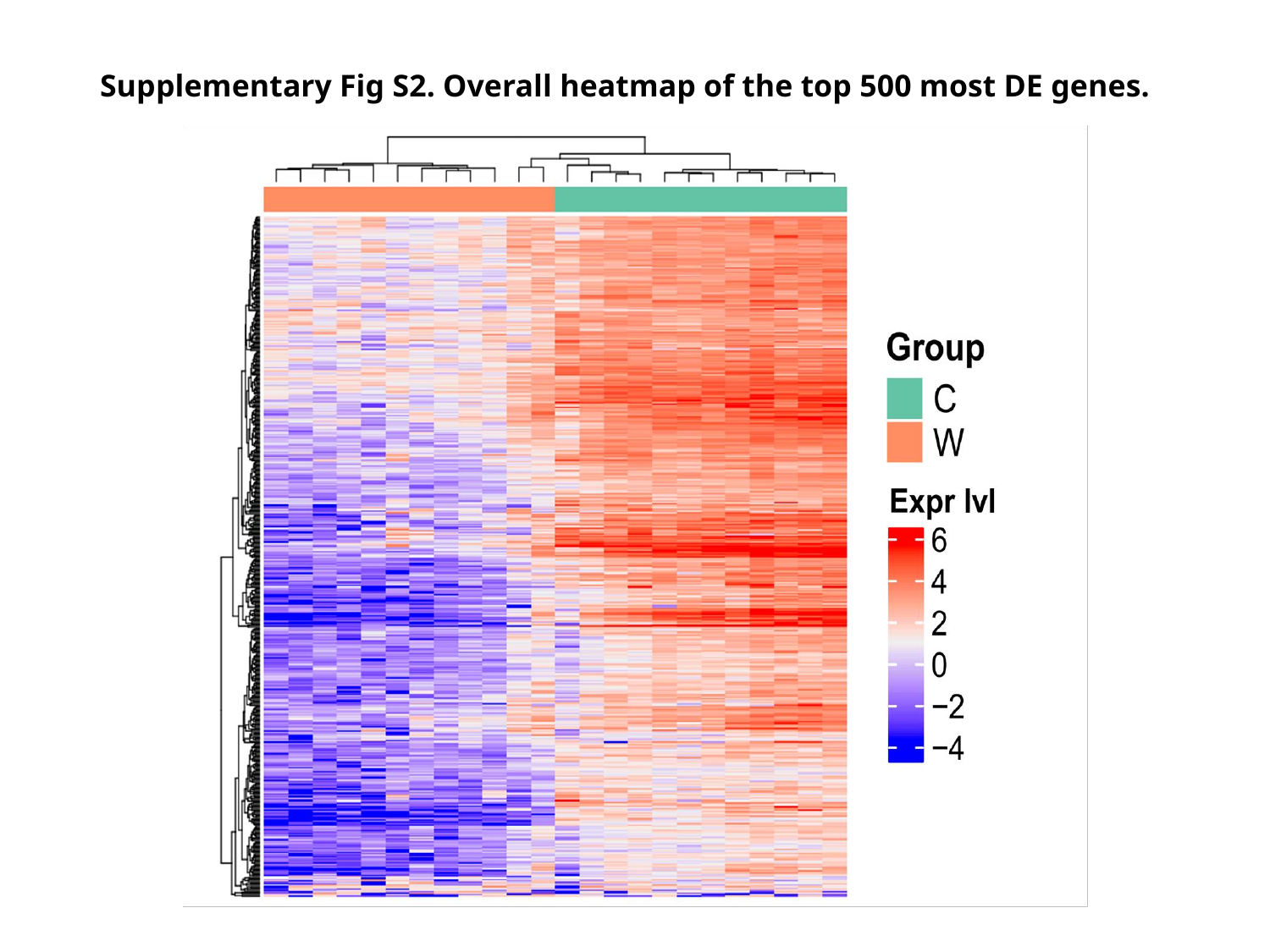

# Supplementary Fig S2. Overall heatmap of the top 500 most DE genes.
