# Supplementary material for: Transcriptome analysis of HPV-induced warts and healthy skin in humans
Source: BMC Med Genomics. 2020 Mar 9;13:35. doi: 10.1186/s12920-020-0700-7 (PMC7063766; doi:10.1186/s12920-020-0700-7)
Supplement: Supplementary file 2 — Additional file 2 Table S1. Top 100 DE genes in warts (FC > 2 and AP < 0.05) as sorted by the quasi-likelihood F test (QLF). [file 12920_2020_700_MOESM2_ESM.docx]

**Table S1. Top 100 DE genes in warts (FC>2 and AP<0.05) as sorted by the quasi-likelihood F test (QLF).**

| Gene ID | Gene name | Gene description | Chr | Expression pattern | QLF | logFC | logCPM | P-value | FDR |
| --- | --- | --- | --- | --- | --- | --- | --- | --- | --- |
| 3868 | KRT16 | keratin 16 | 17 | Up | 378.28 | 7.23 | 13.56 | 2.99 x 10^-17^ | 4.05 x 10^-13^ |
| 255324 | EPGN | epithelial mitogen | 4 | Up | 271.84 | 7.11 | 5.24 | 1.74 x 10^-15^ | 1.02 x 10^-11^ |
| 64137 | ABCG4 | ATP binding cassette subfamily G member 4 | 11 | Up | 254.91 | 5.97 | 5.37 | 3.80 x 10^-15^ | 1.02 x 10^-11^ |
| 6319 | SCD | stearoyl-CoA desaturase | 10 | Up | 254.00 | 3.35 | 8.69 | 3.97 x 10^-15^ | 1.02 x 10^-11^ |
| 8745 | ADAM23 | ADAM metallopeptidase domain 23 | 2 | Up | 250.56 | 4.63 | 5.02 | 4.68 x 10^-15^ | 1.02 x 10^-11^ |
| 388135 | C15orf59 | chromosome 15 open reading frame 59 | 15 | Down | 249.61 | -3.94 | 5.23 | 4.90 x 10^-15^ | 1.02 x 10^-11^ |
| 9517 | SPTLC2 | serine palmitoyltransferase long chain base subunit 2 | 14 | Up | 245.56 | 1.79 | 6.18 | 5.97 x 10^-15^ | 1.02 x 10^-11^ |
| 57823 | SLAMF7 | SLAM family member 7 | 1 | Up | 245.25 | 3.47 | 4.02 | 6.06 x 10^-15^ | 1.02 x 10^-11^ |
| 54809 | SAMD9 | sterile alpha motif domain containing 9 | 7 | Up | 242.97 | 4.33 | 4.92 | 6.78 x 10^-15^ | 1.02 x 10^-11^ |
| 7162 | TPBG | trophoblast glycoprotein | 6 | Up | 233.59 | 2.42 | 6.62 | 1.09 x 10^-14^ | 1.40 x 10^-11^ |
| 8638 | OASL | 2'-5'-oligoadenylate synthetase like | 12 | Up | 232.71 | 5.42 | 6.03 | 1.14 x 10^-14^ | 1.40 x 10^-11^ |
| 1824 | DSC2 | desmocollin 2 | 18 | Up | 230.58 | 5.07 | 7.80 | 1.27 x 10^-14^ | 1.41 x 10^-11^ |
| 2171 | FABP5 | fatty acid binding protein 5 | 8 | Up | 229.42 | 2.71 | 10.24 | 1.35 x 10^-14^ | 1.41 x 10^-11^ |
| 4352 | MPL | MPL proto-oncogene, thrombopoietin receptor | 1 | Up | 225.90 | 5.96 | 4.46 | 1.62 x 10^-14^ | 1.58 x 10^-11^ |
| 2525 | FUT3 | fucosyltransferase 3 (Lewis blood group) | 19 | Up | 223.73 | 3.29 | 4.82 | 1.82 x 10^-14^ | 1.65 x 10^-11^ |
| 26047 | CNTNAP2 | contactin associated protein like 2 | 7 | Up | 221.78 | 5.84 | 3.18 | 2.02 x 10^-14^ | 1.72 x 10^-11^ |
| 642934 | CERNA2 | competing endogenous lncRNA 2 for microRNA let-7b | 10 | Up | 219.91 | 4.20 | 1.87 | 2.24 x 10^-14^ | 1.73 x 10^-11^ |
| 220002 | CYB561A3 | cytochrome b561 family member A3 | 11 | Down | 219.49 | -1.84 | 6.66 | 2.29 x 10^-14^ | 1.73 x 10^-11^ |
| 1452 | CSNK1A1 | casein kinase 1 alpha 1 | 5 | Up | 214.56 | 1.64 | 7.83 | 3.00 x 10^-14^ | 2.14 x 10^-11^ |
| 6317 | SERPINB3 | serpin family B member 3 | 18 | Up | 213.08 | 5.28 | 10.22 | 3.26 x 10^-14^ | 2.21 x 10^-11^ |
| 9388 | LIPG | lipase G, endothelial type | 18 | Up | 211.62 | 4.21 | 3.51 | 3.53 x 10^-14^ | 2.28 x 10^-11^ |
| 2217 | FCGRT | Fc fragment of IgG receptor and transporter | 19 | Down | 206.64 | -2.98 | 5.62 | 4.69 x 10^-14^ | 2.89 x 10^-11^ |
| 3091 | HIF1A | hypoxia inducible factor 1 alpha subunit | 14 | Up | 191.47 | 3.05 | 6.62 | 1.15 x 10^-13^ | 6.79 x 10^-11^ |
| 5376 | PMP22 | peripheral myelin protein 22 | 17 | Down | 187.55 | -2.63 | 6.01 | 1.47 x 10^-13^ | 8.18 x 10^-11^ |
| 57089 | ENTPD7 | ectonucleoside triphosphate diphosphohydrolase 7 | 10 | Up | 187.07 | 2.71 | 4.47 | 1.51 x 10^-13^ | 8.18 x 10^-11^ |
| 8742 | TNFSF12 | TNF superfamily member 12 | 17 | Down | 186.46 | -2.26 | 2.74 | 1.57 x 10^-13^ | 8.18 x 10^-11^ |
| 644945 | KRT16P3 | keratin 16 pseudogene 3 | 17 | Up | 185.90 | 5.13 | 0.85 | 1.63 x 10^-13^ | 8.18 x 10^-11^ |
| 400954 | EML6 | echinoderm microtubule associated protein like 6 | 2 | Up | 180.44 | 2.88 | 3.31 | 2.30 x 10^-13^ | 1.12 x 10^-10^ |
| 4860 | PNP | purine nucleoside phosphorylase | 14 | Up | 178.14 | 2.42 | 7.28 | 2.67 x 10^-13^ | 1.25 x 10^-10^ |
| 6698 | SPRR1A | small proline rich protein 1A | 1 | Up | 175.05 | 5.65 | 10.80 | 3.28 x 10^-13^ | 1.48 x 10^-10^ |
| 6699 | SPRR1B | small proline rich protein 1B | 1 | Up | 173.56 | 4.65 | 11.38 | 3.62 x 10^-13^ | 1.54 x 10^-10^ |
| 6440 | SFTPC | surfactant protein C | 8 | Down | 173.41 | -5.27 | 0.79 | 3.65 x 10^-13^ | 1.54 x 10^-10^ |
| 8548 | BLZF1 | basic leucine zipper nuclear factor 1 | 1 | Up | 173.09 | 1.69 | 4.57 | 3.73 x 10^-13^ | 1.54 x 10^-10^ |
| 285629 | LINC02159 | long intergenic non-protein coding RNA 2159 | 5 | Up | 172.53 | 3.34 | 2.46 | 3.87 x 10^-13^ | 1.55 x 10^-10^ |
| 8991 | SELENBP1 | selenium binding protein 1 | 1 | Down | 171.01 | -2.91 | 4.57 | 4.29 x 10^-13^ | 1.67 x 10^-10^ |
| 594855 | CPLX3 | complexin 3 | 15 | Up | 170.07 | 4.48 | 4.24 | 4.58 x 10^-13^ | 1.72 x 10^-10^ |
| 79631 | EFL1 | elongation factor like GTPase 1 | 15 | Up | 169.68 | 1.36 | 5.14 | 4.70 x 10^-13^ | 1.72 x 10^-10^ |
| 25819 | NOCT | nocturnin | 4 | Up | 169.14 | 2.23 | 3.23 | 4.88 x 10^-13^ | 1.74 x 10^-10^ |
| 3853 | KRT6A | keratin 6A | 12 | Up | 168.15 | 6.81 | 13.91 | 5.22 x 10^-13^ | 1.82 x 10^-10^ |
| 221091 | LRRN4CL | LRRN4 C-terminal like | 11 | Down | 163.00 | -3.92 | 3.45 | 7.46 x 10^-13^ | 2.53 x 10^-10^ |
| 10804 | GJB6 | gap junction protein beta 6 | 13 | Up | 159.97 | 3.06 | 9.35 | 9.26 x 10^-13^ | 3.07 x 10^-10^ |
| 2706 | GJB2 | gap junction protein beta 2 | 13 | Up | 159.42 | 3.40 | 10.34 | 9.63 x 10^-13^ | 3.11 x 10^-10^ |
| 22936 | ELL2 | elongation factor for RNA polymerase II 2 | 5 | Up | 159.04 | 2.21 | 7.59 | 9.90 x 10^-13^ | 3.12 x 10^-10^ |
| 60437 | CDH26 | cadherin 26 | 20 | Up | 158.76 | 4.08 | 4.23 | 1.01 x 10^-12^ | 3.12 x 10^-10^ |
| 283748 | PLA2G4D | phospholipase A2 group IVD | 15 | Up | 158.45 | 3.17 | 8.32 | 1.03 x 10^-12^ | 3.12 x 10^-10^ |
| 9635 | CLCA2 | chloride channel accessory 2 | 1 | Up | 157.89 | 1.78 | 8.66 | 1.08 x 10^-12^ | 3.17 x 10^-10^ |
| 11260 | XPOT | exportin for tRNA | 12 | Up | 157.60 | 1.35 | 6.15 | 1.10 x 10^-12^ | 3.17 x 10^-10^ |
| 1381 | CRABP1 | cellular retinoic acid binding protein 1 | 15 | Down | 157.12 | -4.43 | 2.01 | 1.14 x 10^-12^ | 3.21 x 10^-10^ |
| 5264 | PHYH | phytanoyl-CoA 2-hydroxylase | 10 | Down | 156.28 | -1.93 | 3.68 | 1.21 x 10^-12^ | 3.21 x 10^-10^ |
| 100505839 | SH3PXD2A-AS1 | SH3PXD2A antisense RNA 1 | 10 | Up | 156.21 | 2.00 | 4.57 | 1.22 x 10^-12^ | 3.21 x 10^-10^ |
| 1977 | EIF4E | eukaryotic translation initiation factor 4E | 4 | Up | 156.16 | 1.23 | 4.83 | 1.22 x 10^-12^ | 3.21 x 10^-10^ |
| 1591 | CYP24A1 | cytochrome P450 family 24 subfamily A member 1 | 20 | Up | 156.04 | 5.66 | 2.94 | 1.23 x 10^-12^ | 3.21 x 10^-10^ |
| 6897 | TARS | threonyl-tRNA synthetase | 5 | Up | 154.34 | 1.26 | 6.40 | 1.40 x 10^-12^ | 3.57 x 10^-10^ |
| 101927703 | FOXCUT | FOXC1 upstream transcript | 6 | Down | 153.84 | -5.65 | 1.11 | 1.45 x 10^-12^ | 3.59 x 10^-10^ |
| 118987 | PDZD8 | PDZ domain containing 8 | 10 | Up | 153.79 | 2.27 | 3.91 | 1.45 x 10^-12^ | 3.59 x 10^-10^ |
| 84913 | ATOH8 | atonal bHLH transcription factor 8 | 2 | Down | 152.83 | -2.69 | 2.63 | 1.56 x 10^-12^ | 3.78 x 10^-10^ |
| 286887 | KRT6C | keratin 6C | 12 | Up | 151.86 | 9.15 | 11.91 | 1.68 x 10^-12^ | 3.95 x 10^-10^ |
| 441282 | AKR1B15 | aldo-keto reductase family 1 member B15 | 7 | Up | 151.63 | 3.74 | 2.25 | 1.71 x 10^-12^ | 3.95 x 10^-10^ |
| 9689 | BZW1 | basic leucine zipper and W2 domains 1 | 2 | Up | 151.56 | 2.17 | 6.87 | 1.72 x 10^-12^ | 3.95 x 10^-10^ |
| 139886 | SPIN4 | spindlin family member 4 | X | Up | 150.97 | 3.61 | 2.24 | 1.79 x 10^-12^ | 4.06 x 10^-10^ |
| 101928987 | LOC101928987 | uncharacterized LOC101928987 | 4 | Up | 150.48 | 2.64 | 1.19 | 1.86 x 10^-12^ | 4.12 x 10^-10^ |
| 3671 | ISLR | immunoglobulin superfamily containing leucine rich repeat | 15 | Down | 150.01 | -2.57 | 4.92 | 1.93 x 10^-12^ | 4.12 x 10^-10^ |
| 4140 | MARK3 | microtubule affinity regulating kinase 3 | 14 | Up | 149.84 | 1.30 | 6.44 | 1.95 x 10^-12^ | 4.12 x 10^-10^ |
| 5718 | PSMD12 | proteasome 26S subunit, non-ATPase 12 | 17 | Up | 149.65 | 1.37 | 5.21 | 1.98 x 10^-12^ | 4.12 x 10^-10^ |
| 29966 | STRN3 | striatin 3 | 14 | Up | 149.58 | 1.92 | 4.71 | 1.99 x 10^-12^ | 4.12 x 10^-10^ |
| 100507420 | LINC01605 | long intergenic non-protein coding RNA 1605 | 8 | Up | 149.52 | 3.26 | 2.79 | 2.00 x 10^-12^ | 4.12 x 10^-10^ |
| 400451 | FAM174B | family with sequence similarity 174 member B | 15 | Down | 148.82 | -2.30 | 2.55 | 2.11 x 10^-12^ | 4.26 x 10^-10^ |
| 54845 | ESRP1 | epithelial splicing regulatory protein 1 | 8 | Up | 148.68 | 1.27 | 7.77 | 2.13 x 10^-12^ | 4.26 x 10^-10^ |
| 554202 | MIR31HG | MIR31 host gene | 9 | Up | 147.98 | 5.68 | 2.09 | 2.25 x 10^-12^ | 4.43 x 10^-10^ |
| 201895 | SMIM14 | small integral membrane protein 14 | 4 | Up | 147.64 | 1.66 | 4.20 | 2.31 x 10^-12^ | 4.48 x 10^-10^ |
| 56300 | IL36G | interleukin 36, gamma | 2 | Up | 147.23 | 3.68 | 8.69 | 2.38 x 10^-12^ | 4.56 x 10^-10^ |
| 768211 | RELL1 | RELT like 1 | 4 | Down | 146.50 | -1.59 | 4.08 | 2.52 x 10^-12^ | 4.76 x 10^-10^ |
| 140576 | S100A16 | S100 calcium binding protein A16 | 1 | Up | 145.43 | 1.33 | 9.27 | 2.74 x 10^-12^ | 5.03 x 10^-10^ |
| 51458 | RHCG | Rh family C glycoprotein | 15 | Up | 145.42 | 8.75 | 8.54 | 2.74 x 10^-12^ | 5.03 x 10^-10^ |
| 11197 | WIF1 | WNT inhibitory factor 1 | 12 | Down | 144.84 | -7.68 | 5.00 | 2.87 x 10^-12^ | 5.20 x 10^-10^ |
| 5266 | PI3 | peptidase inhibitor 3 | 20 | Up | 143.94 | 8.92 | 11.35 | 3.08 x 10^-12^ | 5.50 x 10^-10^ |
| 201799 | TMEM154 | transmembrane protein 154 | 4 | Up | 143.36 | 1.98 | 6.91 | 3.23 x 10^-12^ | 5.69 x 10^-10^ |
| 1475 | CSTA | cystatin A | 3 | Up | 142.66 | 2.46 | 11.02 | 3.41 x 10^-12^ | 5.93 x 10^-10^ |
| 114794 | ELFN2 | extracellular leucine rich repeat and fibronectin type III domain containing 2 | 22 | Down | 142.41 | -5.24 | 0.99 | 3.48 x 10^-12^ | 5.97 x 10^-10^ |
| 3073 | HEXA | hexosaminidase subunit alpha | 15 | Down | 141.13 | -1.54 | 5.28 | 3.85 x 10^-12^ | 6.53 x 10^-10^ |
| 57016 | AKR1B10 | aldo-keto reductase family 1 member B10 | 7 | Up | 140.51 | 2.91 | 6.12 | 4.04 x 10^-12^ | 6.77 x 10^-10^ |
| 3487 | IGFBP4 | insulin like growth factor binding protein 4 | 17 | Down | 140.17 | -2.52 | 6.88 | 4.15 x 10^-12^ | 6.88 x 10^-10^ |
| 5275 | SERPINB13 | serpin family B member 13 | 18 | Up | 139.90 | 3.36 | 8.15 | 4.25 x 10^-12^ | 6.95 x 10^-10^ |
| 5210 | PFKFB4 | 6-phosphofructo-2-kinase/fructose-2,6-biphosphatase 4 | 3 | Up | 139.40 | 1.75 | 3.94 | 4.42 x 10^-12^ | 7.14 x 10^-10^ |
| 4644 | MYO5A | myosin VA | 15 | Up | 139.19 | 1.88 | 6.45 | 4.50 x 10^-12^ | 7.18 x 10^-10^ |
| 55064 | SPATA6L | spermatogenesis associated 6 like | 9 | Up | 138.40 | 2.04 | 3.40 | 4.79 x 10^-12^ | 7.56 x 10^-10^ |
| 5194 | PEX13 | peroxisomal biogenesis factor 13 | 2 | Up | 138.20 | 1.35 | 4.25 | 4.87 x 10^-12^ | 7.60 x 10^-10^ |
| 140606 | SELENOM | selenoprotein M | 22 | Down | 137.95 | -2.18 | 3.21 | 4.97 x 10^-12^ | 7.67 x 10^-10^ |
| 262 | AMD1 | adenosylmethionine decarboxylase 1 | 6 | Up | 136.79 | 1.52 | 6.25 | 5.46 x 10^-12^ | 8.33 x 10^-10^ |
| 4267 | CD99 | CD99 molecule (Xg blood group) | X | Down | 136.26 | -1.67 | 7.02 | 5.71 x 10^-12^ | 8.61 x 10^-10^ |
| 29904 | EEF2K | eukaryotic elongation factor 2 kinase | 16 | Down | 135.96 | -2.28 | 7.31 | 5.85 x 10^-12^ | 8.73 x 10^-10^ |
| 7474 | WNT5A | Wnt family member 5A | 3 | Up | 135.02 | 3.29 | 5.67 | 6.32 x 10^-12^ | 9.33 x 10^-10^ |
| 23266 | ADGRL2 | adhesion G protein-coupled receptor L2 | 1 | Up | 134.46 | 2.40 | 6.02 | 6.63 x 10^-12^ | 9.62 x 10^-10^ |
| 29780 | PARVB | parvin beta | 22 | Down | 134.39 | -2.13 | 3.73 | 6.66 x 10^-12^ | 9.62 x 10^-10^ |
| 51011 | FAHD2A | fumarylacetoacetate hydrolase domain containing 2A | 2 | Down | 133.45 | -1.40 | 3.81 | 7.21 x 10^-12^ | 1.03 x 10^-9^ |
| 10855 | HPSE | heparanase | 4 | Up | 133.38 | 2.31 | 5.98 | 7.25 x 10^-12^ | 1.03 x 10^-9^ |
| 374918 | IGFL1 | IGF like family member 1 | 19 | Up | 132.79 | 7.96 | 4.72 | 7.62 x 10^-12^ | 1.04 x 10^-9^ |
| 147463 | ANKRD29 | ankyrin repeat domain 29 | 18 | Up | 132.51 | 1.96 | 3.19 | 7.80 x 10^-12^ | 1.04 x 10^-9^ |
| 57214 | CEMIP | cell migration inducing hyaluronan binding protein | 15 | Up | 132.50 | 2.67 | 5.38 | 7.80 x 10^-12^ | 1.04 x 10^-9^ |
| 7123 | CLEC3B | C-type lectin domain family 3 member B | 3 | Down | 132.35 | -3.46 | 5.56 | 7.90 x 10^-12^ | 1.04 x 10^-9^ |

*logFC*: base 2 logarithm of fold change; *logCPM*: base 2 logarithm of counts per million; *QLF*: quasi-likelihood F test; *FDR*: false discovery rate or adjusted p-value; *Chr*: chromosome.
