# Supplementary material for: Transcriptome analysis of HPV-induced warts and healthy skin in humans
Source: BMC Med Genomics. 2020 Mar 9;13:35. doi: 10.1186/s12920-020-0700-7 (PMC7063766; doi:10.1186/s12920-020-0700-7)
Supplement: Supplementary file 3 — Additional file 3 Table S2. Functional annotation clustering of the DE genes only showing DE genes with a p-value < 0.05. BP stands for biological processes, MF for molecular function, CC for cellular component, and GO for gene ontology. [file 12920_2020_700_MOESM3_ESM.docx]

**Table S2: Functional annotation clustering of the DE genes only showing DE genes with a p-value < 0.05.** BP stands for biological processes, MF for molecular function, CC for cellular component, and GO for gene ontology.

|  | **Category** | **Term** | **P-value** | **Genes** |
| --- | --- | --- | --- | --- |
| Annotation Cluster 1  Enrichment Score: 4.85 | CC | GO:0001533~cornified envelope | 2.12 x 10^-6^ | HRNR, LCE3A, LCE3D, ANXA1, SPRR2G, SPRR2F, SPRR2E, CDSN, CST6, SPRR1A, SPRR2D, SPRR1B, SPRR2A, RPTN, SPRR2B, CNFN, TGM1, LCE3E, CSTA, IVL, PRR9 |
|  | BP | GO:0031424~keratinization | 5.97 x 10^-6^ | HRNR, LCE3A, LCE3D, SPRR2G, SPRR2F, SFN, SPRR2E, KRT17, SPRR1A, SPRR2D, KRT16, SPRR1B, SPRR2A, SPRR2B, CNFN, TGM1, TGM3, KRT2, LCE3E, IVL, ABCA12 |
|  | BP | GO:0030216~keratinocyte differentiation | 1.89 x 10^-5^ | WNT5A, LCE3A, S100A7, LCE3D, SPRR2G, TP63, SPRR2F, SPRR2E, CERS3, CDSN, SPRR2D, SPRR2A, TGM1, SPRR2B, TGM3, YAP1, IVL, PRR9, ADAM9, TXNIP, ANXA1, EPHA2, SPRR1A, KRT16, SPRR1B, LCE3E, CSTA |
|  | BP | GO:0018149~peptide cross-linking | 1.69 x 10^-4^ | LCE3A, F13A1, LCE3D, ANXA1, SPRR2G, SPRR2F, SPRR2E, SPRR1A, SPRR2D, SPRR1B, SPRR2A, SPRR2B, TGM1, TGM3, LCE3E, CSTA, IVL, PRR9, FN1 |
| Annotation Cluster 2  Enrichment Score: 3.70 | BP | GO:0019882~antigen processing and presentation | 1.12 x 10^-6^ | HLA-DQB1, RAB3B, HLA-DRB1, HLA-A, HLA-C, FCGRT, HLA-B, CTSS, HLA-DMB, HLA-DQA2, CD74, PSMB8, RAET1E, RAET1G, CD209, RAB34, ULBP2, RAET1L, HLA-DRB5, HLA-DPA1, HLA-DPB1, RAB10, CTSH, HLA-DRA |
|  | BP | GO:0060333~interferon-gamma-mediated signaling pathway | 1.29 x 10^-6^ | HLA-DQB1, HLA-DQB2, HLA-DRB1, OAS3, OAS1, OAS2, B2M, CAMK2D, HLA-DRB5, CAMK2B, HLA-DPB1, CAMK2A, CIITA, ICAM1, HCK, HLA-A, HLA-C, HLA-B, MID1, STAT1, HLA-DQA2, HLA-F, OASL, IRF7, HLA-DPA1, IRF4, GBP2, HLA-DRA |
|  | CC | GO:0042613~MHC class II protein complex | 1.77 x 10^-6^ | HLA-DQB1, HLA-DQB2, HLA-DRB1, HLA-A, HLA-C, HLA-DMB, HLA-DMA, HLA-DQA2, CD74, HLA-DRB5, HLA-DPA1, HLA-DPB1, HLA-DOA, HLA-DRA |
|  | MF | GO:0032395~MHC class II receptor activity | 6.72 x 10^-6^ | HLA-DQB1, HLA-DQB2, KRT17, HLA-DRB1, HLA-C, HLA-DPA1, HLA-DPB1, HLA-DOA, HLA-DMA, HLA-DQA2, HLA-DRA |
|  | CC | GO:0030669~clathrin-coated endocytic vesicle membrane | 2.62 x 10^-5^ | WNT5A, HLA-DQB1, HLA-DQB2, TYRP1, LDLR, HLA-DRB1, FZD5, HLA-DQA2, CD74, AP1S3, CD207, ROR2, HLA-DRB5, HBEGF, HLA-DPA1, CLVS1, HLA-DPB1, HLA-DRA |
|  | BP | GO:0002504~antigen processing and presentation of peptide or polysaccharide antigen via MHC class II | 3.54 x 10^-5^ | HLA-DQB1, HLA-DQB2, HLA-DRB1, HLA-DRB5, HLA-DPA1, HLA-DPB1, HLA-DMB, HLA-DOA, HLA-DMA, HLA-DQA2, HLA-DRA |
|  | BP | GO:0019886~antigen processing and presentation of exogenous peptide antigen via MHC class II | 2.59 x 10^-4^ | KIF23, HLA-DQB1, HLA-DQB2, KIF4A, SEC24A, HLA-DRB1, HLA-DMB, HLA-DMA, CD74, AP1S3, KIFAP3, FCER1G, HLA-DRB5, HLA-DPB1, HLA-DOA, KIF2A, SEC23A, DYNC1I1, KIF11, KIF15, KIF18A, CENPE, CTSS, HLA-DQA2, CTSV, HLA-DPA1, HLA-DRA, CTSF |
|  | MF | GO:0042605~peptide antigen binding | 2.97 x 10^-4^ | HLA-DQB1, HLA-DRB1, CD209, TAP1, HLA-A, HLA-DRB5, HLA-C, HLA-DPA1, FCGRT, HLA-B, HLA-DPB1, HLA-DRA, HLA-F |
|  | CC | GO:0071556~integral component of lumenal side of endoplasmic reticulum membrane | 4.13 x 10^-4^ | HLA-DQB1, HLA-DQB2, HLA-DRB1, HLA-A, HLA-DRB5, HLA-C, HLA-DPA1, HLA-B, HLA-DPB1, HLA-DQA2, CD74, HLA-DRA, HLA-F |
|  | CC | GO:0012507~ER to Golgi transport vesicle membrane | 7.72 x 10^-4^ | HLA-DQB1, SEC23A, HLA-DQB2, SEC24A, HLA-DRB1, HLA-A, HLA-C, HLA-B, HLA-DQA2, CD74, HLA-F, B2M, MCFD2, HLA-DRB5, HLA-DPA1, AREG, HLA-DPB1, HLA-DRA |
|  | MF | GO:0023026~MHC class II protein complex binding | 9.06 x 10^-4^ | HSP90AA1, HLA-DRB1, CD81, HLA-DMB, HLA-DOA, HLA-DMA, CD74, HSPA8, HLA-DRA |
|  | CC | GO:0032588~trans-Golgi network membrane | 0.014 | HLA-DQB1, HLA-DQB2, HLA-DRB1, MYO1B, SLC24A5, HLA-DQA2, CD74, USP6NL, AP1S3, KIF13A, RAB31, TMEM165, HLA-DRB5, HLA-DPA1, CALN1, CLVS1, CLIP3, HLA-DPB1, TMEM79, HLA-DRA, RHOBTB3 |
|  | CC | GO:0030658~transport vesicle membrane | 0.017 | HLA-DQB1, HLA-DQB2, CLCN3, ARFGEF3, HLA-DRB1, HLA-DRB5, CA4, HLA-DPA1, HLA-DPB1, HLA-DQA2, CD74, HLA-DRA |
|  | BP | GO:0031295~T cell costimulation | 0.017 | HLA-DQB1, HLA-DQB2, HLA-DRB1, EFNB3, LGALS1, CCL19, TNFRSF14, HLA-DQA2, VAV1, CD86, CCL21, PIK3CA, HLA-DRB5, HLA-DPA1, CD4, HLA-DPB1, CD24, YES1, SPN, HLA-DRA |
|  | BP | GO:0002503~peptide antigen assembly with MHC class II protein complex | 0.027 | HLA-DRB1, HLA-DMB, HLA-DMA, HLA-DRA |
| Annotation Cluster 3  Enrichment Score: 2.08 | MF | GO:0004674~protein serine/threonine kinase activity | 0.003 | CCNT2, CDK17, PRPF4B, TTK, CASK, AURKA, MAP3K5, OSR1, SLK, MAP3K9, EEF2K, PIK3CA, STK39, TLK1, MASTL, CAB39, ANKK1, IRAK2, CDK1, PHKG1, PRKCI, PKN1, PRKCH, WNK2, MARK3, SRPK1, CDKL5, MARK1, DAPK1, PRKCB, MAP4K4, MAST4, MAP4K5, MAPK6, RIOK3, HIPK3, HIPK2, TESK1, NEK8, BMP2K, CAMK1, BUB1B, CSNK1G3, RIPK4, EIF2AK2, MELK, NEK7, CAMK1D, KALRN, MAP4K1, CHEK1, STK17A, STK32C, VRK2, STK32A, SBK1, SPEG, MAP3K1, BUB1, CAMK2D, CAMK2B, DYRK3, STK38L, CAMK2A, DCLK1, AATK, CSNK1A1, ALPK3, TAOK1, OXSR1, RPS6KA5, PLK4, PLK2, MAPK14, BMPR1B, CDK20 |
|  | BP | GO:0006468~protein phosphorylation | 0.006 | CCNT2, CDK17, PRPF4B, CASK, AURKA, FER, BTK, CCNE1, MAP3K5, MAP3K9, COQ8A, PIK3CA, RARA, STK39, TLK1, ANKK1, IRAK2, ADAM10, PHKG1, STK26, PRKCI, PKN1, PRKCH, WNK2, SRPK1, CDKL5, MARK1, DAPK1, PRKCB, MAP4K4, CCND1, MAP4K5, MAPK6, SCYL2, RIOK3, HIPK3, HIPK2, NEK8, BMP2K, BUB1B, CAMK1, WNT11, RIPK4, EIF2AK2, CTSG, NEK7, CAMK1D, KALRN, FGFR1, CCL2, FGR, PPP4R1, MAP4K1, STK17A, EPHB6, VRK2, SBK1, SPEG, MORC3, MAP3K1, BUB1, ZAP70, CAMK2D, CAMK2B, DYRK3, STK38L, CAMK2A, RUNX3, DCLK1, AATK, CSNK1A1, PDK1, ALPK3, TAOK1, HCK, PDK4, NPR1, OXSR1, GAS6, GMFB, RPS6KA5, PLK4, PLK2, RASSF2, ADM2, BMPR1B, CDK20, IGFBP3 |
|  | MF | GO:0004672~protein kinase activity | 0.029 | CDK17, PRPF4B, CASK, AURKA, BTK, MAP3K5, MAP3K9, COQ8A, EEF2K, STK39, ANKK1, CDK1, PHKG1, STK26, PRKCI, PKN1, PRKCH, PKDCC, SRPK1, CDKL5, DAPK1, PRKCB, MAP4K4, CCND1, STYK1, MAP4K5, SCYL2, HIPK3, HIPK2, TESK1, NEK8, BMP2K, PDGFRA, BUB1B, CAMK1, ROR2, CSNK1G3, RIPK4, EIF2AK2, MELK, NEK7, KALRN, CDK5R1, CCL2, MAP4K1, CHEK1, STK17A, EPHB6, VRK2, MAP3K1, BUB1, CAMK2B, DYRK3, CAMK2A, STK38L, DCLK1, AATK, CSNK1A1, PDK1, TAOK1, MAP2K4, PDK4, NPR1, RPS6KA5, MAPK14, RASSF2, CDK20 |
| Annotation Cluster 4  Enrichment Score: 2.02 | MF | GO:0017147~Wnt-protein binding | 8.99 x 10^-4^ | SMO, FZD10, SFRP1, SFRP2, TRABD2B, ROR2, WIF1, WLS, FRZB, FZD5, APCDD1, FZD7, FZD6 |
|  | BP | GO:0035567~non-canonical Wnt signaling pathway | 0.03 | WNT5A, SMO, SFRP2, FRZB, FZD5, WNT7A, FZD6 |
|  | MF | GO:0042813~Wnt-activated receptor activity | 0.033 | SMO, FZD10, SFRP1, SFRP2, FRZB, FZD5, FZD7, FZD6 |
